# The trap. When Marco Decorpeliada strikes back

**DOI:** 10.1017/S2045796021000317

**Published:** 2021-06-09

**Authors:** Baptiste Brun

**Affiliations:** Rennes 2 University, Rennes, Bretagne, France

**Keywords:** DSM 5, Art Brut, Outsider Art, Epidemiology

## The story

In Spring 2010, the Parisian viewers were astonished by a very strange exhibition held in La Maison Rouge. This regretted contemporary art place located near la Bastille used to present together so-called Outsider and insider artists without any distinction. There, people discovered displayed in showcases the *Schizomètres* made by a French named Marco Decorpeliada (Decorpeliada, 2010a, 2010b).

Decorpeliada was born in 1947 in Morocco. After his father's death, he came to Ozoir-la-Ferrière near Paris with his mother and his two sisters. For a few years, he studied medicine without any success. Then he travelled a lot for 10 years. In 1995, back in France, he began to live a period of wandering and suffered several psychic crisis. After his mother's death, one of those led him to a long period of internment in different psychiatric hospitals. In these institutions – La Timone, Marseilles; Maison Blanche and Sainte-Anne, Paris; Le Vinatier, Lyon –, he was diagnosed with paranoid schizophrenia (20.0) regarding DSM-IV nomenclature. Moreover, several additional diagnoses have been pronounced on him such as social anxiety disorder (40.1), frotteuristic disorder (65.8), sibling relational problem (93.3) and so on. After making a large bunch of objects around 2004 – the ones viewers could admire at La Maison Rouge in 2010 –, he started to travel again. He disappeared in 2006 in a plane crash near Leticia, Colombia.

At the beginning of the 2000s, Sven Legrand, one of his psychiatrists, realised that Decorpeliada was persecuted by the process of diagnosis he was subjected to. Receptive to his patient's sensibility to art – Decorpeliada used to visit exhibitions and liked to read about art and artists – Legrand encouraged him to develop his inclination. In parallel to this, Decorpeliada begun to make unique objects. Legrand discovered them during their conversation. As a matter of fact, although closely studying the three-digit codes of the Diagnostic and Statistical Manual of Mental Disorders, Decorpeliada had noticed that the DSM-IV codes were identical to those of items attached to the Picard frozen food catalogue, the French market leader in frozen food products. Therefore, he systematically underlined correspondences between the two registers. Hence, ‘labelled’ amongst other things as having paranoid personality disorders (60.0) by psychiatrists, Marco Decorpeliada countered the diagnosis with ‘60.0 Pre-fried Hash Browns XL’ and so on: to catatonic type schizophrenia (20.1), he responded ‘20.1, whole cooked shrimps’; to frotteuristic disorder (65.8), ‘65.8 Four beef *pavé* in the tenderloin’. The discovery was a revelation to him. Indeed he used to eat items of the frozen food catalogue that correspond to diagnosis he was subject to. Using an Excel table, a printer, scissors and glue, he illustrated the hundreds of possible correspondences to the nearest millimetre on what he called the *Schizomètres*: those were carpenter's rulers on which the patient glued small printed labels. Marco Decorpeliada's *bricolages* were some kind of a trick to visually fast check correspondences. Then, he made new objects. Collecting ten freezer's doors in trash dumps – one door for each of the ten main classes of the DSM –, Decorpeliada covered them with a hundred boxes grid. He divided each grid in two parts regarding the same process of correspondences: on the first one, he wrote DSM diagnosis items in black on the white surface of the freezer's doors; on the second one, he wrote the name of a frozen food item in white on the black surface of magnets fixed in regard. Then, he observed that to some items chosen either in DSM-IV or in Picard frozen food catalogue, there were gaps in correspondences. As Decorpeliada wrote it to Sven Legrand in an email dated the 20th December 2004: ‘But I must admit that you are right, it is absolutely true, there are diseases that are missing. I am convinced of this since I have eaten all Picard products containing scallops. You will necessarily agree with me that all products containing scallops have scallops in common, and you will not take away from me that all the diseases corresponding to these Picard products must have a link between them since these products all contain the same thing: scallops. I could also have taken the example of button mushrooms, there are plenty of them at Picard. Well, there are items with scallops that don't match. For example, 48.0 scallops Argentina without coral, or 21.0 scallops Japan with coral, or 92.0 scallop plate, don't correspond to anything in the DSM. It is incredible that those in the DSM ignore diseases, unless they hide them, but the proof is there’ (Decorpeliada, 2010a, 2010b).

**Fig. 1.**
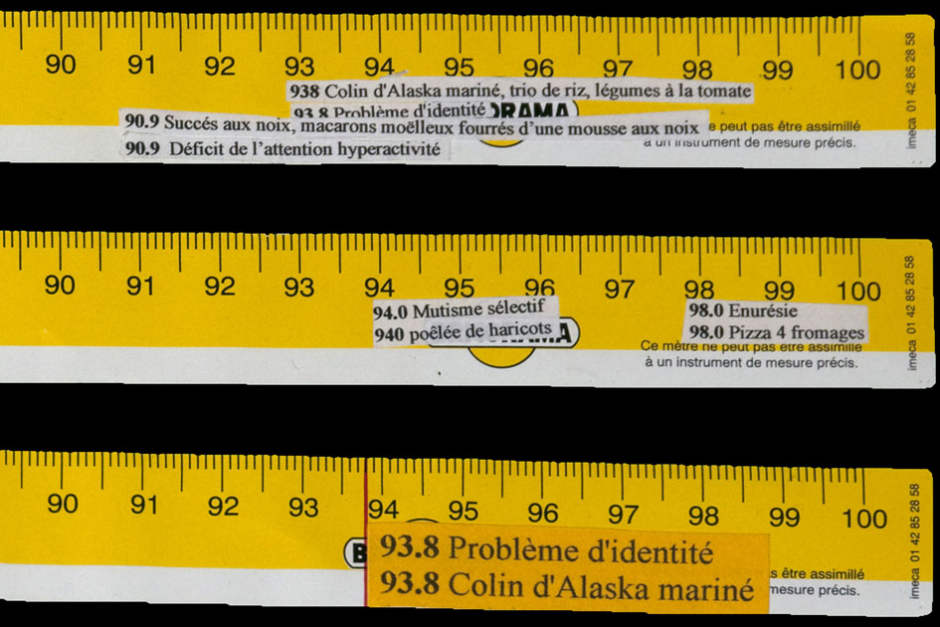
Marco Decorpeliada, Three schizomètres (details), ca 2004, private collection.

**Fig. 2.**
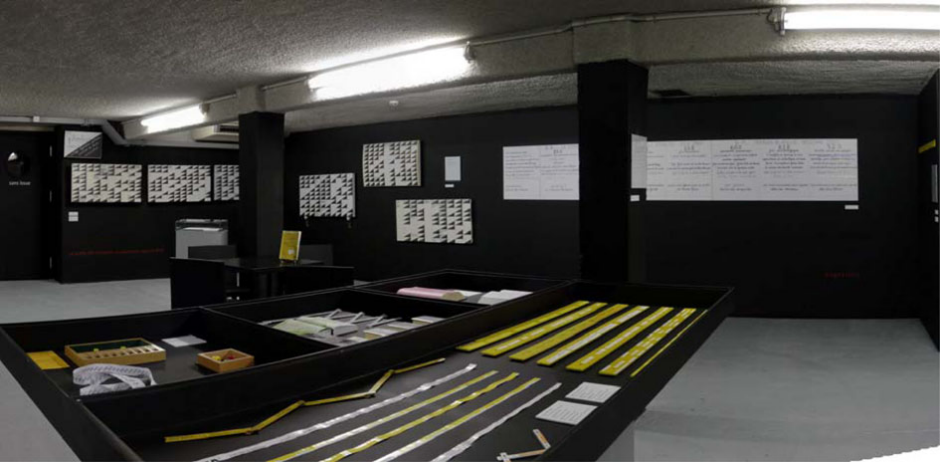
Exhibition view of “Marco Decorpeliada. Schizomètres”, Paris, La maison rouge, Feb. 19 – May 16 2010.

The thrill is sensible. In Decorpeliada's opinion, the ‘proof’ fulfils his sense of persecution, the very structure of his paranoid structure: ‘those in the DSM’ cannot be so ignorant! More broadly, Decorpeliada's freezer's doors show with evidence that the DSM is just a tool with its own deficiencies and lacks. In a Michel Foucault's perspective, it is obvious that this discovery manifests the historical dimension of every taxonomies.

## The trap

May the reader of *Epidemiology and Psychiatric Sciences* forgive me, but this story is a hoax. Everything is true – the exhibition, the objects shown, the correspondences between DSM-IV and Picard frozen food catalogue – but Marco Decorpeliada. His figure was invented by four psychoanalysts (Jacques Adam, Laurent Cornaz, Dominique de Liège and Yan Pélissier) and by the writer Marcel Bénabou of the French literary group *L'Oulipo*. Marco Decorpeliada is an acronym created from the syllables of their name. This creation and its ‘revelation’ were made to put into criticism uses and abuses of DSM-IV (Bâton, 2015; Diener, 2017; Cabañas, 2018).

When the *EPS* journal asked me for a contribution dedicated to an Outsider artist, I have decided to devote it to Decorpeliada. When I discovered his work, I was very excited even if I had suspected it was a prank. At the time I was writing my PhD dissertation dedicated to the work of the French painter Jean Dubuffet, focusing on his activity dedicated to Art Brut afterwar (Brun, 2019). Processes of categorisation in art's worlds – according to Howard Becker's sociological perspective – were part of my reflections. Decorpeliada's objects, and especially the *Schizomètres* and fridge doors, underline the assumption of the omnipotence of the viewer with Art Brut or Outsider Art. They manifest how the amateur's gaze is deeply shaped by a certain understanding of those notions and forms and storytelling related to them in terms of biographies and to a certain romantic understanding. What the viewer generally expects of Outsider Art is deeply rooted to an expressionist even primitivistic horizon. Art Brut is, in a way, realised in the game of relations which is established between the producer, the work and the one which qualifies it as Outsider Art. This game is conditioned by the history of taste. Such a production made in a psychiatric hospital or an object shaped by a peasant will be qualified in turn as a clinical document (in the case of productions resulting from the psychiatric field) or as a toy or the result of a playful tinkering (in the case of productions resulting e.g. from a rural environment), before being celebrated as Outsider Art. These differences in status are intimately linked to the history of contemporary art in 20th and 21st centuries. Some objects of the Collection of the Psychiatric Hospital of Heidelberg seen by Hans Prinzhorn in the 1920s as symptomatic of a pathology – but not worthy of the qualification ‘art’ that could allow them to be published in his seminal book of 1922, *Bildnerei der Geisteskranken* – will be shown several decades later as belonging to the artistic field, thanks to the glance of the collector, the critic or the curator. Contemporary art shapes our gaze. Thus, Antoine de Galbert, the former creator of La Maison Rouge, a great collector ‘trapped’ by Marco Decorpeliada whose objects echo a post-minimalist aesthetic that challenged a renewal of Outsider's repertoire of forms. Despite the disappointment, Galbert decided to dedicate an exhibition to "Marco's case".

Marco Decorpeliada institutes two processes of downgrading, one operating in the field of psychopathology, the other one in the field of art. He encourages us to show the constructed character of Art Brut/Outsider Art, regarding the topic of historicity, like an aesthetic category operating within the art's world. In this perspective, it's possible to think about it as a collective fantasy that has been naturalised for ideological purposes, a contemporary myth (Barthes, 1957) that adapts itself to fashion and tendencies, freeing itself from its political, sociological and historical substratum. Therefore, it needs to be questioned again and again, for each of its metamorphosis. More broadly, Marco Decorpeliada and his work act as a specular image revealing the historical and cultural character of all classifications (Vidal, 2018), from the DSM to Art Brut.

## About the author

Baptiste Brun is codirector of the Department of History of Art and Archeology at the University of Rennes 2, France. His research focuses on the interactions between the worlds of art, psychiatry and ethnography since the post-war period. He is a member of various editorial boards (*Gradhiva*, *Perspective*, *Critique d'art*). He wrote several articles dedicated to Contemporary Art and, especially, the field of Outsider Art. He co-edited the edition of the unpublished manuscript of the *Almanach de l'Art Brut* (2016). Among other publications he is the author of *Jean Dubuffet et la besogne de l'Art Brut: critique du primitivisme* (2019). In 2019-2020, he curated the exhibition *Jean Dubuffet, un Barbare en Europe* (Marseilles, Mucem; IVAM, Valencia; Geneva, MEG).

Carole Tansella, *Section Editor*

## References

[ref1] Barthes R (1957) Mythologies. Paris: Seuil.

[ref2] Bâton D (2015) Schizomètres. In Fol C (ed.), Poetry that Breaks Through the Diagnosis Barrier, in Outsider Art in Question. Bruxelles: CFC editions.

[ref3] Brun B (2019) Jean Dubuffet et la Besogne de L'Art Brut. Critique du Primitivisme. Dijon: Les presses du réel.

[ref4] Cabañas KM (2018) Learning from Madness. Brazilian Modernism and Global Contemporary Art. Chicago: Chicago Press University.

[ref5] Decorpeliada M (2010a) Schizomètre. Petit Manuel de Survie en Milieu Psychiatrique. Paris: EPEL.

[ref6] Decorpeliada M (2010b) Coulisses. Paris: the author.

[ref7] Diener Y (2017) Conférence art brut ou art conceptuel? Les deux, mon capitaine. *Charlie Hebdo*, n. 1294, Paris.

[ref8] Vidal B (2018) L'effet Schizomètre. Quand L'art Brut Dégivre la Psychopathologie. Paris: EPEL.

